# Study on the Competitive Substitution of Four Polyphenolic Compounds on the HSA-Bound α-Zearalenol In Vitro Simulated Modeling

**DOI:** 10.3390/toxins18010007

**Published:** 2025-12-22

**Authors:** Cheng Chen, Lu Chen, Hongyuan Zhou, Xiao Li Shen, Liang Ma

**Affiliations:** 1 School of Public Health, Zunyi Medical University, Zunyi 563000, China; 2 College of Food Science, Southwest University, Chongqing 400715, Chinazhouhy@swu.edu.cn (H.Z.); 3 Chongqing Key Laboratory of Specialty Food Co-Built by Sichuan and Chongqing, Chongqing 400715, China

**Keywords:** α-zearalenol, polyphenolic compounds, human serum albumin, binding constant, competitive substitution

## Abstract

α-Zearalenol (α-ZOL), the primary metabolite of zearalenone (ZEN), is a prevalent mycotoxin in agricultural products (e.g., corn, wheat) and poses health risks due to its toxicity. However, strategies to mitigate its toxicity are needed. Therefore, this study aims to determine whether selected polyphenols (quercetin, baicalin, rosmarinic acid, naringenin) can competitively displace α-ZOL from human serum albumin (HSA) and to clarify the interaction mechanisms. The results showed that competitive interactions between α-ZOL, HSA, and the polyphenols were observed. The polyphenols bound HSA more tightly than α-ZOL (higher *K_a_*) and significantly reduced α-ZOL’s *K_a_*, indicating direct competition. Moreover, as evidenced by synchronous fluorescence, the polyphenols altered the microenvironments of tyrosine and tryptophan residues, directly impacting α-ZOL binding. The HPLC-ultrafiltration results revealed that the polyphenols tested competitively displaced α-ZOL from HSA, with the relative potency of quercetin ≈ baicalin > rosmarinic acid > naringenin. Collectively, our competitive binding assays demonstrate that quercetin, baicalin, rosmarinic acid, and naringenin competitively displace α-ZOL from its binding site(s) on HSA. Thus, our study not only suggests a novel mechanism to alleviate the toxicity of ZEN and α-ZOL but also provides a scientific basis for developing dietary interventions against these mycotoxins.

## 1. Introduction

Polyphenolic compounds were recognized as major bioactive constituents of functional foods with both medicinal and nutritional relevance. They were widely distributed in fruits, vegetables, legumes, and cereals, and had attracted considerable scientific attention owing to their natural origin and favorable safety profiles [1]. Accumulating evidence demonstrated that representative polyphenols, including quercetin, baicalin, rosmarinic acid, and naringenin, exerted diverse biological activities, such as antioxidant, anti-inflammatory, cardioprotective, immunomodulatory, and anticancer effects [2,3]. With the rapid expansion of the health and wellness industry, polyphenols have been recognized not only for their nutritional value but also for their potential roles in disease prevention and intervention. Therefore, they were considered essential bioactive substances supporting the concept of “preventive treatment of disease” and delaying the onset of chronic disorders [4,5]. Therefore, systematic studies on the functional properties of structurally representative polyphenols and their interactions with key serum and tissue proteins are essential for developing functional foods and promoting public health.

Zearalenone (ZEN) was identified as a mycotoxin with strong estrogenic activity, primarily produced by Fusarium species. It was able to competitively bind to mammalian estrogen receptors, disrupt estrogen metabolism, and consequently induce reproductive toxicity [6,7]. α-Zearalenol (α-ZOL), one of the primary metabolites of ZEN, had also been detected in human biological samples. Gratz et al. reported the presence of both ZEN and α-ZOL in urinary samples collected from children (*n* = 21) in the United Kingdom during a mycotoxin exposure assessment [8]. Previous studies demonstrated that the estrogenic activity of α-ZOL was approximately three to four times higher than that of its parent compound, ZEN [9]. In addition, α-ZOL exhibited slightly greater polarity than ZEN, which facilitated its persistence through multiple stages of food processing, thereby increasing the risk of food contamination and posing potential hazards to both human and animal health [10].

Human serum albumin (HSA), the predominant carrier protein in the human body, accounted for more than 50% of plasma proteins. Once absorbed into the bloodstream, small bioactive molecules were able to reversibly bind to HSA [11]. It has been well established that HSA plays a crucial role in the pharmacokinetics and toxicokinetics of various endogenous and exogenous substances, in which competitive binding often occurs between different drugs or toxins that share the same binding sites on the protein [12,13]. In recent years, increasing attention has been paid to the modulation of toxin-induced toxicity through interactions with small bioactive or pharmaceutical molecules, providing a potential strategy for in vivo detoxification. Poór et al. [14] reported that several flavonoids, such as galangin and quercetin, share the same binding sites on HSA with ochratoxin A (OTA). These compounds could competitively displace OTA from the HSA-OTA complex, thereby facilitating its release and subsequent elimination. Similarly, Ma et al. [15] demonstrated that quercetin, a representative flavonol, competes with aflatoxin B1 (AFB1) for binding to HSA, leading to a decrease in the level of HSA-bound AFB1 in vivo. Furthermore, Grosse et al. [16] found that vitamin E mitigated the inhibitory effect of ZEN on DNA synthesis by competing for the same binding site, thus alleviating its toxicity. In addition, curcumin was reported to bind to subdomain IIA of HSA, suggesting that nutritional intervention might lower the binding affinity of α-ZOL to HSA and accelerate its clearance [17]. Given the pivotal role of HSA in the metabolism of both endogenous and exogenous substances, the interactions between HSA and small molecular compounds have drawn increasing scientific interest.

Therefore, in this study, four structurally representative polyphenolic compounds—quercetin (Que), baicalin (Bai), rosmarinic acid (RA), and naringenin (NG)—which share the same binding site (subdomain IIA) on HSA as α-ZOL [18,19,20,21], were selected to investigate their competitive binding interactions with α-ZOL-bound to HSA. The primary objective was to evaluate their potential to displace HSA-bound α-ZOL, thereby reducing the overall toxin burden within the organism. This study established an in vitro model that simulates reduced toxin residence time in biological systems, thereby decreasing potential toxicity and providing a theoretical basis for applying polyphenols in health promotion and disease prevention.

## 2. Results

### 2.1. Competitive Binding of Polyphenols and α-ZOL at the HSA Binding Site

**Figure 1:**
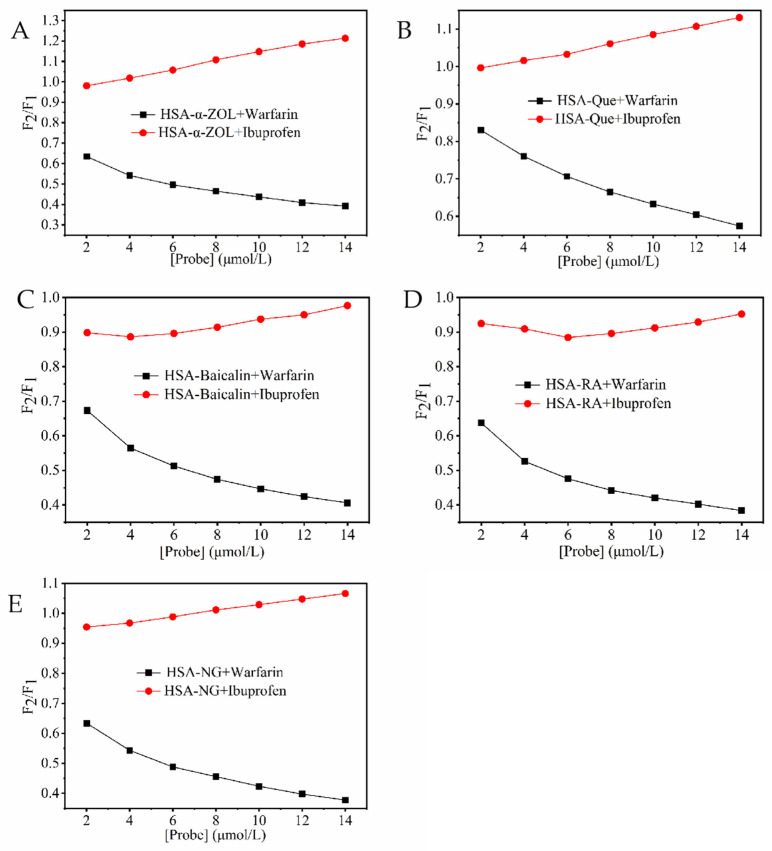
Effect of probe type on the fluorescence intensities of HSA-ligand systems. (**A**) (HSA-α-ZOL) + Warfarin/Ibuprofen; (**B**) (HSA-Que) + Warfarin/Ibuprofen; (**C**) (HSA-Baicalin) + Warfarin/Ibuprofen; (**D**) (HSA-RA) + Warfarin/Ibuprofen; (**E**) (HSA-NG) + Warfarin/Ibuprofen. The concentrations of Warfarin/Ibuprofen were 0~12 μmol/L.

Warfarin and ibuprofen were employed as site markers for binding sites I and II of human serum albumin (HSA), respectively. As shown in Figure 1, the gradual addition of warfarin significantly decreased the fluorescence intensity of both the HSA-α-ZOL complex and the complexes formed between HSA and various polyphenolic ligands. In contrast, the fluorescence intensity of the HSA-ligand complexes exhibited no significant change upon incremental addition of ibuprofen. These results indicated that both α-ZOL and the tested polyphenols primarily compete with warfarin for binding at site I of HSA. Therefore, it was inferred that the four polyphenolic compounds shared the same binding site on HSA as α-ZOL, suggesting a potential mechanism of competitive displacement.

### 2.2. Effects of Polyphenolic Compounds on the HSA-α-ZOL System

**Figure 2:**
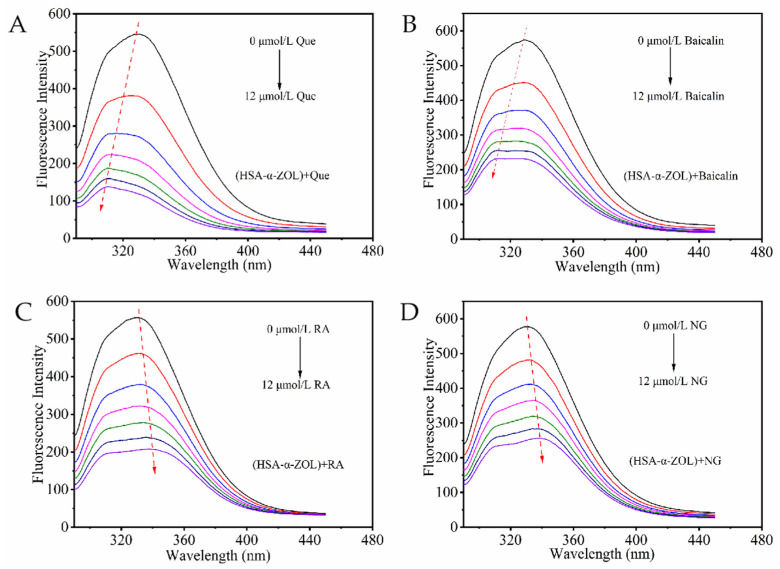
Fluorescence quenching of polyphenolic compounds on HSA-α-ZOL system. (**A**) (HSA-α-ZOL) + Que. (**B**) (HSA-α-ZOL) +Baicalin. (**C**) (HSA-α-ZOL) + RA. (**D**) (HSA-α-ZOL) + NG. The red arrows indicate the trend of the protein maximum emission peak shifting toward longer or shorter wavelengths. The concentrations of polyphenols were 0, 2, 4, 6, 8, 10, and 12 μmol/L from top to bottom.

Human serum albumin (HSA) contained endogenous fluorescent amino acid residues including tryptophan (Trp), tyrosine (Tyr), and phenylalanine (Phe), which produced strong fluorescence emission when excited at 280 nm [22]. In the HSA-α-ZOL system, when both HSA and α-ZOL concentrations were maintained at 2 μmol/L, quercetin, baicalin, rosmarinic acid, and naringenin were successively added at concentrations of 0, 2, 4, 6, 8, 10, and 12 μmol/L. The fluorescence spectra of the resulting mixtures were subsequently recorded (Figure 2). As the concentrations of the polyphenolic compounds increased, the fluorescence intensity of HSA within the HSA-α-ZOL system gradually decreased in a concentration-dependent manner, suggesting that these polyphenolic compounds altered the secondary structure of HSA and formed stable complexes with it [23]. Furthermore, after the addition of polyphenolic compounds, distinct blue shifts (Figure 2A,B) or red shifts (Figure 2C,D) were observed in the fluorescence emission peaks of the system. These observations indicated that polyphenolic compounds interacted with HSA in the HSA-α-ZOL system, thereby inducing alterations in the microenvironment surrounding the Trp and Tyr residues within the protein.

### 2.3. Competitive Binding of Polyphenolic Compounds and α-ZOL to HSA

The fluorescence quenching of proteins was generally categorized as either static or dynamic quenching. Static quenching occurred when the quencher formed a non-fluorescent ground-state complex with the fluorophore. Dynamic quenching resulted from diffusion-driven collisions between the quencher and the excited fluorophore, leading to reduced fluorescence intensity [23]. The Stern–Volmer equation was applied to calculate the quenching rate constant (*K_sv_*) of the system, thereby elucidating the binding mechanism involved [10].

(1) $$\frac{F_{0}}{F}=1+K_{\mathrm{sv}}Q=1+{\;K}_{q}\tau_{0}Q$$

In this equation, F_0_ and F represented the fluorescence intensities of the protein in the absence and presence of the quencher, respectively; *K_q_* denotes the biomolecular quenching rate constant (L/mol·s); τ_0_ referred to the average fluorescence lifetime of the fluorophore in the absence of the quencher (typically 10^−8^ s) [24]; *K_sv_* represented the Stern–Volmer quenching constant (L/mol); and *Q* indicated the concentration of the quencher (mol/L).

By plotting *F*_0_/*F* versus *Q*, the Stern–Volmer equation was linearly fitted, and the correlation coefficients HSA were all greater than 0.9900, as presented in Table 1. In all systems, the calculated *K_q_* values ranged from 10^12^ to 10^13^ L/mol·s, which were significantly higher than the maximum dynamic collision quenching rate constant (2.0 × 10^10^ L/mol·s). These results indicated that both the quenching of HSA by α-ZOL and the quenching of the HSA-α-ZOL complex by polyphenolic compounds proceeded via a static quenching mechanism, implying the formation of non-fluorescent ground-state complexes [25].

A dynamic equilibrium was typically reached in small molecule–macromolecule interactions, with some small molecules bound to the protein and others remaining free in solution. Accordingly, the Lineweaver–Burk static quenching equation was applied to evaluate the binding parameters.

(2) $$\mathrm{Log}\frac{\left(F\;-\;F_{0}\right)}{F}=\;\log K_{a}+n\log Q$$

In this equation, *F*_0_ and *F* represented the fluorescence intensities of the protein in the absence and presence of the quencher, respectively; *K_a_* was the binding constant; *n* denoted the number of binding sites per protein molecule; and *Q* was the ligand concentration.

Generally, the binding constant (*K_a_*) reflected the stability of the interaction between a ligand and a protein. A larger *K_a_* value indicated a stronger and more stable interaction, whereas a smaller value suggested weaker binding and lower stability [14]. As shown in Table 1, the number of binding sites between α-ZOL and HSA was approximately one, indicating that the optimal binding ratio between HSA and α-ZOL was about 1:1. Given that the polyphenolic compounds examined in this study shared the same binding site (subdomain IIA) on HSA as α-ZOL, a competitive binding mechanism was plausible. In the competitive systems where α-ZOL and HSA were mixed at a 1:1 molar ratio, the addition of equimolar concentrations of quercetin, baicalin, rosmarinic acid, or naringenin resulted in a significant increase in the binding constants. These results suggested that the tested polyphenolic compounds exerted a pronounced competitive displacement effect toward α-ZOL on HSA.

Furthermore, the influence of binding order between polyphenols and α-ZOL on competitive displacement was evaluated. The substitutive potential of polyphenols toward α-ZOL was further verified. The displacement capacity of α-ZOL was examined in preformed HSA-polyphenol complexes. The results are presented in Table 2. The order of addition of the polyphenolic compounds and α-ZOL did not alter the quenching mechanism; all systems still exhibited static quenching, resulting from the formation of non-fluorescent ground-state complexes [25]. Based on the calculated binding constants, a marked reduction in the affinity of α-ZOL for HSA was observed in the presence of polyphenolic compounds. Competitive displacement of HSA-bound polyphenolic compounds by α-ZOL was not observed. Moreover, the variations in *K_a_* values among different polyphenolic compounds followed the same trend as their competitive displacement capacities shown in Table 1, further confirming the above conclusion.

### 2.4. Effects of Polyphenolic Compounds on the Microenvironment of Amino Acid Residues in the HSA-α-ZOL System

**Figure 3:**
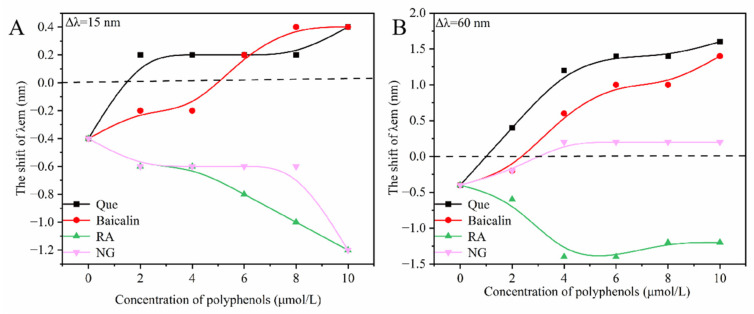
The effects of polyphenolic compounds on the microenvironment of Tyr and Trp in the system of HSA-α-ZOL. (**A**) the synchronous fluorescence spectrum at Δλ = 15 nm. (**B**): the synchronous fluorescence spectrum at Δλ = 60 nm. The concentrations of polyphenols were 0, 2, 4, 6, 8, and 10 μmol/L. Que, Baicalin, RA and NG stand for quercetin, baicalin, rosmarinic acid and naringenin, respectively.

Synchronous fluorescence spectroscopy (Δλ = 15 nm for tyrosine and Δλ = 60 nm for tryptophan) was employed to probe changes in the microenvironment of amino acid residues. Shifts in the maximum emission wavelength indicated alterations in the polarity surrounding these residues [26]. As shown in Figure 3, the addition of different polyphenolic compounds to the HSA-α-ZOL system altered the polarity around both tyrosine and tryptophan residues. In the absence of polyphenols, α-ZOL alone induced a slight blue shift (−0.4 nm), suggesting reduced polarity and increased hydrophobicity in the vicinity of these residues. In contrast, red shifts were observed for quercetin and baicalin. Increased polarity and reduced hydrophobicity were indicated. The microenvironment around Tyr and Trp residues was modified, and conformational changes in HSA were induced. Rosmarinic acid, however, led to a blue shift, implying enhanced hydrophobicity, likely due to interactions with hydrophilic amino acid regions. Naringenin produced a blue shift for tyrosine but a red shift for tryptophan, reflecting different interaction distances with these residues [27]. Overall, the hydrophobic enhancement around tyrosine residues was more pronounced than that around tryptophan residues.

### 2.5. Competitive Displacement of α-ZOL by Polyphenolic Compounds

To further confirm the competitive displacement ability of quercetin, baicalin, rosmarinic acid, and naringenin toward α-ZOL bound to HSA, the molecular weight difference between HSA (66.5 kDa) and α-ZOL was utilized. Free α-ZOL was separated through 10 kDa ultrafiltration centrifuge tube based on molecular sieving, and its concentration was determined by HPLC. The difference in α-ZOL content between the experimental and control groups was calculated as the amount of α-ZOL displaced from HSA by the polyphenolic compounds.

As shown in Table 3, the competitive displacement rate of α-ZOL bound to HSA increased significantly with the rising concentrations of polyphenolic compounds, showing a positive correlation. Among them, quercetin and baicalin exhibited the strongest displacement ability, followed by rosmarinic acid and naringenin, consistent with the binding constant trends observed in the HSA-α-ZOL systems. A single binding site on HSA (n ≈ 1) was engaged by α-ZOL and the polyphenols. Complete replacement of α-ZOL at equimolar ratios was not achieved by the polyphenols. Competitive displacement was influenced by multiple interaction forces and the spatial configuration of each molecule. Further studies were therefore required to elucidate the detailed mechanism underlying this competitive interaction.

## 3. Discussion

The toxicokinetics of mycotoxins are largely governed by their reversible binding to human serum albumin (HSA), which critically regulates their plasma distribution, metabolic availability, and elimination rates [14,28]. Consequently, the competitive displacement of toxin–albumin complexes by dietary polyphenols has been proposed as a potential nutritional detoxification strategy. Such displacement may transiently increase the free toxin fraction in plasma, thereby facilitating tissue uptake, enzymatic metabolism, and subsequent excretion, ultimately shortening the effective plasma half-life [29,30]. This concept is supported by previous findings; for instance, curcumin can displace α-zearalenol from HSA, thereby reducing its toxicity [17]. Similarly, the strong binding of other emerging mycotoxins to HSA underscores the critical role of albumin association in modulating their toxicokinetics [31]. Collectively, these reports suggest that compounds capable of displacing mycotoxins from serum albumin may serve as functional modulators of mycotoxin bioavailability rather than simple binders.

Physiologically, displacement of α-ZOL from HSA is expected to transiently increase its unbound plasma fraction [17,32]. α-ZOL is rapidly biotransformed in vivo, mainly through reductive metabolism followed by phase II conjugation, including glucuronidation and sulfation [33]. An increased free fraction may therefore accelerate metabolic clearance and subsequent biliary or urinary excretion [17,34]. Importantly, HSA-ligand binding is highly dynamic and reversible, with association and dissociation occurring on a seconds-to-minutes timescale under physiological conditions. Thus, α-ZOL released from HSA can rapidly access metabolic pathways, while also retaining the capacity to efficiently rebind to albumin at downstream vascular sites, depending on local concentrations, blood flow, and competing ligands. Overall, the net toxicological outcome reflects a balance among competitive displacement, metabolic turnover, and rapid rebinding equilibria [34].

Our study elucidates the molecular mechanism by which specific polyphenols—quercetin, baicalin, rosmarinic acid, and naringenin—compete with α-ZOL for HSA binding. Fluorescence quenching experiments revealed that α-ZOL forms a stable ground-state complex with HSA via static quenching, with rate constants indicating strong binding within the hydrophobic pocket of subdomain IIA. The addition of polyphenols induced comparable static quenching, suggesting competition at the same or overlapping sites. These observations are in agreement with previous reports describing competitive interactions between flavonoids and small-molecule toxins at Sudlow’s site I [17,32]. This was corroborated by synchronous fluorescence spectroscopy, which showed polyphenol-induced changes in the microenvironment of tyrosine and tryptophan residues. Red and blue shifts in emission maxima tracked alterations in local polarity and hydrophobicity, linking structural features like planarity and hydroxyl substitution to binding dynamics.

Site-marker displacement assays confirmed that both α-ZOL and the polyphenols primarily bind to Sudlow’s site I. The marked decrease in the binding constant (*K_a_*) of α-ZOL in the presence of polyphenols directly confirms competitive displacement. The displacement efficacy, verified quantitatively by ultrafiltration-HPLC, followed the order: quercetin ≈ baicalin > rosmarinic acid > naringenin. This trend aligns with the known binding mode of flavonoids in subdomain IIA, which involves π–π stacking and hydrogen bonding with residues such as Tyr150, Trp214, and Arg222 [19,20,21]. The superior efficacy of quercetin and baicalin can be attributed to their polyhydroxylated, planar skeletons, which favor extensive interactions with the binding pocket. In contrast, the simpler framework of naringenin and the partial hydrophilicity of rosmarinic acid limit their affinity, highlighting the critical roles of molecular planarity and hydrophobicity.

In conclusion, our findings demonstrate that a “multi-hydroxylated planar structure with a hydrophobic core” is a key determinant for the competitive displacement of toxins from serum albumin. Beyond establishing a molecular basis for polyphenol–HSA competition, these results suggest a plausible toxicokinetic mechanism by which dietary polyphenols may transiently enhance mycotoxin clearance in vivo. However, given the rapid metabolism of polyphenols themselves and the dynamic nature of albumin binding equilibria, future studies should integrate in vivo toxicokinetic analyses and explore synergistic effects in composite formulations or nanocarrier systems to optimize stability, competitive efficiency, and biological relevance.

## 4. Conclusions

Our results establish that quercetin, baicalin, rosmarinic acid, and naringenin competitively displace α-ZOL from HSA. This process is initiated by the alteration of key amino acid residue microenvironments and results in static fluorescence quenching. The order of displacement efficacy was quantified as quercetin ≈ baicalin > rosmarinic acid > naringenin by ultrafiltration-HPLC. Collectively, these findings elucidate a molecular mechanism by which dietary polyphenols can reduce the bioavailability of α-ZOL, thereby providing a scientific basis for novel dietary strategies aimed at mitigating mycotoxin toxicity.

## 5. Materials and Methods

### 5.1. Reagents

Human serum albumin (HSA, purity ≥ 99%) was purchased from Sigma-Aldrich (St. Louis, MO, USA). α-Zearalenol (α-ZOL, purity ≥ 99%) was obtained from Pribolab (Singapore). Quercetin, rosmarinic acid, and naringenin (purity ≥ 97%) were supplied by Aladdin Reagent Co., Ltd. (Shanghai, China), and baicalin (purity ≥ 99%) was obtained from Macklin Biochemical Co., Ltd. (Shanghai, China). Amicon Ultra-15 centrifugal filter units (10 kDa) were purchased from Millipore (Millipore, Billerica, MA, USA). A reverse-phase HPLC column (150 mm × 4.6 mm, 5 μm) was obtained from Agilent Technologies (Santa Clara, CA, USA). Chromatographic-grade acetonitrile and methanol were supplied by Honeywell (Charlotte, NC, USA). All other reagents were of analytical grade.

HSA was dissolved in Tris–HCl buffer (0.05 mol/L, pH 7.4) to prepare a 40 μmol/L stock solution. α-ZOL and each polyphenolic compound were dissolved in chromatographic-grade acetonitrile to prepare 1 mmol/L stock solutions. They were diluted to the desired concentrations immediately before use.

### 5.2. Fluorescence Quenching Spectroscopy

Fluorescence measurements were performed using an F-2500 fluorescence spectrophotometer under the following conditions: excitation wavelength, 280 nm; emission wavelength, 290–450 nm; scanning speed, 240 nm·min^−1^; excitation and emission slit widths, 5 nm; gain, 3; excitation voltage, 400 V.

To eliminate the effect of the quencher on the fluorescence spectra, the measured fluorescence intensity was corrected according to the following equation to minimize the inner filter effect [35].

$$F_{\mathrm{cor}}=F_{\mathrm{obs}}\;\times {\;e}^{\frac{A_{\mathrm{ex}}+A_{\mathrm{em}}}{2}}$$

where *F_obs_* and *F_cor_* represent the observed and corrected fluorescence intensities, respectively, and *A_ex_* and *A_em_* denote the absorbances of the ligand at the excitation and emission wavelengths of HSA.

### 5.3. Synchronous Fluorescence Spectroscopy

At 298 K, synchronous fluorescence spectra of the HSA-α-ZOL system (2.0 μmol/L, 1:1 molar ratio) were recorded with wavelength intervals (Δλ) of 15 nm and 60 nm. Polyphenolic compounds (1.0 mmol/L stock solutions) were added to achieve final concentrations of 2, 4, 6, 8, and 10 μmol/L. Each mixture was equilibrated for 3 min prior to scanning.

### 5.4. Site Marker Competitive Binding Assay

Binding-site competition studies were conducted using warfarin (site I marker) and ibuprofen (site II marker). 2.0 μmol/L HSA solution was prepared, and α-ZOL or each polyphenolic compound was added at a molar ratio of 2:1 (ligand:HSA). Warfarin or ibuprofen was then added to achieve final concentrations ranging from 0 to 12.0 μmol/L. The percentage of α-ZOL or polyphenol displaced by the site marker was calculated according to the following equation [22]:

$$\mathrm{Displacement}\;=\;\frac{F_{2}}{F_{1}}\;\times \;100\%$$

where *F*_1_ and *F*_2_ represent the fluorescence intensities of the HSA-ligand complex in the absence and presence of the site markers, respectively.

### 5.5. Determination of Competitive Displacement Ratio

For ultrafiltration studies, 40 μmol/L HSA and α-ZOL were mixed at a 1:1 molar ratio, followed by the addition of polyphenolic compounds at final concentrations of 20, 40, and 80 μmol/L (molar ratios of 1:1:0.5, 1:1:1, and 1:1:2, respectively). The mixtures were ultrafiltered through 10 kDa centrifugal filters at 10,000 rpm and 4 °C for 15 min to obtain the free α-ZOL fraction, followed by washing with Tris–HCl buffer (pH 7.4).

The wash solutions were collected, evaporated under nitrogen at 45 °C, and redissolved in 1 mL of the mobile phase (methanol/water, 65:35, *v*/*v*) for HPLC analysis.

A blank control containing only 40 μmol/L α-ZOL and a control group containing HSA-α-ZOL without polyphenols were prepared in parallel. The competitive displacement ratio of polyphenols for α-ZOL was calculated as follows:

$$\mathrm{Displacement}\;\mathrm{ratio}\;\left(\%\right)\;=\;\frac{m_{\mathrm{sample}}-m_{\mathrm{control}}}{m_{\mathrm{blank}\;}}\times 100\%$$

### 5.6. HPLC Analysis

α-ZOL was quantified using a reverse-phase C18 column (4.6 × 150 mm, 5 μm) under the following chromatographic conditions: mobile phase, methanol/water (65:35, *v*/*v*); flow rate, 0.80 mL·min^−1^; injection volume, 100 μL; column temperature, 25 °C. The fluorescence detector was set at an excitation wavelength of 315 nm and an emission wavelength of 455 nm. Under the described HPLC conditions, α-ZOL was detected at a retention time of approximately 7.7 min, with minor variations depending on column performance and system conditions.

Each sample was analyzed within 20 min. A calibration curve was constructed by linear regression of peak area (*y*) versus α-ZOL concentration (*x*, 0.0214–4.72 μg/mL), yielding the equation *y* = 2,169,590*x* + 85,205 (R^2^ = 0.9991), indicating excellent linearity. The limit of detection (LOD) and limit of quantification (LOQ) were 0.001 μg/mL and 0.003 μg/mL, respectively (at S/N ratios of 3:1 and 10:1).

Recovery tests within the same linear range yielded 94.24–98.49%. This demonstrates that ultrafiltration had minimal impact on α-ZOL quantification and is suitable for assessing the competitive displacement effects of polyphenolic compounds.

### 5.7. Data Analysis

All experiments were performed in triplicate, and the results were expressed as mean ± standard deviation (SD). Parallel data were processed using Microsoft Excel 2019, and statistical analyses were conducted with SPSS statistics 29.0 (IBM, Armonk, NY, USA). 

## Figures and Tables

**Table 1:** List of quenching constants *K_sv_*, quenching rate constant *K_q_*, binding constants *K_a_*, and binding-site number *n* of polyphenolic substances for the system of HSA-α-ZOL.

Systems	*K_sv_* (×10^5^ L/moL)	*K_q_* (×10^13^ L/moL·s)	*K_a_* (×10^4^ L/moL)	*n*
HSA-α-ZOL	1.91	1.91	5.25	0.9288
(HSA-α-ZOL) + Que	2.14	2.14	39.91	0.9440
(HSA-α-ZOL) + Baicalin	1.01	1.01	48.62	1.0903
(HSA-α-ZOL) + RA	0.65	0.65	15.37	0.9899
(HSA-α-ZOL) + NG	0.76	0.76	7.39	0.9163

**Table 2:** List of quenching constants *K_sv_*, quenching rate constant *K_q_*, binding constants *K_a_*, and binding-site number n of α-ZOL for the system of HSA-polyphenol.

Systems	*K_sv_* (×10^5^ L/moL)	*K_q_* (×10^13^ L/mol·s)	*K_a_* (×10^4^ L/moL)	*n*
HSA-α-ZOL	1.91	1.91	5.25	0.9288
(HSA-Que) + α-ZOL	0.79	0.79	0.60	0.7954
(HSA-Baicalin) + α-ZOL	1.61	1.61	0.67	0.7458
(HSA-RA) + α-ZOL	1.82	1.82	1.76	0.8135
(HSA-NG) + α-ZOL	1.01	1.01	1.84	0.8766

**Table 3:** Competitive substitution rates of polyphenolic compounds on α-ZOL in different systems.

Concentration Ratios	Systems			
	HSA:α-ZOL:Que	HSA:α-ZOL:Baicalin	HSA:α-ZOL:RA	HSA:α-ZOL:NG
1:1:0.5	24.12 ± 3.40% ^a^	22.71 ± 1.33% ^a^	20.36 ± 1.15% ^b^	18.27 ± 1.12% ^c^
1:1:1	35.08 ± 5.22% ^a^	33.12 ± 1.61% ^a^	30.29 ± 0.38% ^b^	27.21 ± 0.05% ^c^
1:1:2	54.21 ± 4.52% ^a^	52.44 ± 1.07% ^a^	49.93 ± 1.98% ^b^	40.41 ± 0.76% ^c^

Note: Different lowercase letters within the same column indicate significant differences (*p* < 0.05).

## Data Availability

The original contributions presented in this study are included in the article. Further inquiries can be directed to the corresponding author.
